# Evaluating Marine Cyanobacteria as a Source for CNS Receptor Ligands

**DOI:** 10.3390/molecules23102665

**Published:** 2018-10-17

**Authors:** Andrea L. Rague, Stacy-Ann J. Parker, Kevin J. Tidgewell

**Affiliations:** Department of Medicinal Chemistry, Graduate School of Pharmaceutical Sciences, Duquesne University Pittsburgh, PA 15282, USA; raguea@duq.edu (A.L.R.); sannjparker@gmail.com (S.-A.J.P.)

**Keywords:** marine cyanobacteria, G-protein coupled receptor, central nervous system, natural products

## Abstract

Natural products have a long history as a source of psychoactive agents and pharmacological tools for understanding the brain and its circuitry. In the last two decades, marine cyanobacteria have become a standard source of natural product ligands with cytotoxic properties. The study of cyanobacterial metabolites as CNS modulatory agents has remained largely untapped, despite the need for new molecules to treat and understand CNS disorders. We have generated a library of 301 fractions from 37 field collected cyanobacterial samples and screened these fractions against a panel of CNS receptors using radiolabeled ligand competitive-binding assays. Herein we present an analysis of the screening data collected to date, which show that cyanobacteria are prolific producers of compounds which bind to important CNS receptors, including those for 5-HT, DA, monoamine transporters, adrenergic, sigma, and cannabinoid receptors. In addition to the analysis of our screening efforts, we will also present the isolation of five compounds from the same cyanobacterial collection to illustrate how pre-fractionation followed by radioligand screening can lead to rapid identification of selective CNS agents. The systematic screening of natural products sources, specifically filamentous marine cyanobacteria, will yield a number of lead compounds for further development as pharmacological tools and therapeutics.

## 1. Introduction

Since the 1970s [1,2], marine cyanobacteria have been studied as a source for novel bioactive natural products. The high degree of chemical diversity among cyanobacterial natural products is attributed to their complex biosynthetic machinery and relatively large genomes that integrates important secondary metabolite pathways with polyketide synthases (PKS) and nonribosomal peptide synthases (NRPS). The broad spectrum of marine cyanobacterial natural products translates into an array of biological activities, including but not limited to, anticancer [3], antibacterial [4], antimalarial [5] and anti-inflammatory [6] activities. 

Due to some limitations, such as difficulty of collection, inadequate sample sizes, or general toxicity of some extracts, the biological diversity of marine ecosystems has not been widely studied as a source of modulators of G-protein coupled receptors (GPCRs) and the central nervous system (CNS). Nevertheless there are a number of interesting leads which have been discovered based on structural similarity to endogenous neurotransmitters [7]. There are known links between CNS disorders and neurotransmitter signaling through specific receptors [8,9,10]. Because of this, there is an opportunity to explore marine cyanobacteria for leads targeting these receptors to treat associated diseases. Our previous work [11,12] has shown in vivo activity in models of depression and anxiety from marine cyanobacterial extracts and fractions targeting serotonin receptors (5-HTRs). By utilizing a broad GPCR screen we believe additional leads can be discovered but hits must be followed up with additional in vitro and in vivo assays examining specificity, efficacy, and toxicity. In addition to 5-HT, cyanobacterial fractions with affinity for a number of other therapeutically relevant receptors have been discovered, including the σ_1_ and σ_2_ receptors, which have been hypothesized to be targets for the treatment of neuropathic pain [13].

Cyanobacterial natural products possess a diversity in structural types from peptidic compounds, to a variety of lipophilic, polyketide derived molecules and compounds of mixed biosynthetic origin [14]. Although cyanobacteria have the ability to produce alkaloids and carbohydrates, these classes are far less common in cyanobacteria than in plants or fungi [15]. Secondary metabolites produced by marine cyanobacteria share structural similarities and motifs with endogenous ligands of GPCRs and therefore could mimic their binding to receptors. Beyond orthosteric interactions, these cyanobacterial metabolites have the potential to act as allosteric and protein-protein interaction modulators. 

As part of our research to discover CNS ligands we have screened extracts from cyanobacterial collections from Panama and Curacao for their ability to bind to 5-HTRs, DARs, MATs, and other CNS receptors using the NIMH Psychoactive Drug Screening Program (PDSP) [16]. Herein, we report an analysis of the results of this effort and discuss the isolation and characterization of five compounds from a single collection, a known cyclic depsipeptide, veraguamide C (**1**), new fatty acid-derived ester, compound **2**, along with compound **3** [17], propenediester [18] (**4**) and grenadadiene [19] (**5**) as a case study to illustrate the effectiveness of this method in some fractions and highlighting the need for method improvement.

## 2. Cyanobacterial Library Screening

### 2.1. Radiolabaled Ligand Screening of Marine Cyanobacterial Extracts and Fractions

**Figure 1:**
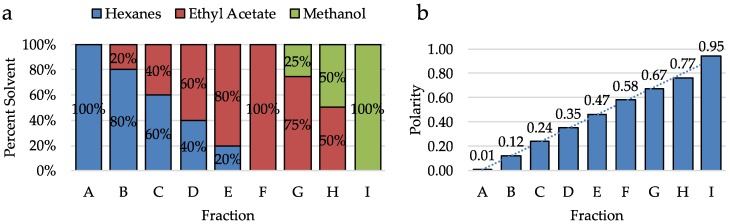
(**a**) Solvent system used to elute each fraction for crude fractionation. Solvent system starts with 100% hexanes for fraction A and increases in polarity through fraction I (100% methanol). (**b**) Polarity of each solvent system used for crude fractionation. The polarity of solvents increases linearly from fractions A through I.

Prior to screening, cyanobacterial samples are extracted exhaustively in a solution of dichloromethane and methanol, then the extract is subjected to column chromatography using the solvent step gradient illustrated in Figure 1 to create nine fractions. The polarities of the solvents in this step gradient increase linearly with each fraction. A sample of each fraction and the crude is prepared and sent to the NIMH Psychoactive Drug Screening Program (PDSP) for screening against a panel of ~50 receptor including 5-HTRs, DARs, MATs, σRs, and other CNS receptors.

From the extracts of 37 cyanobacterial field collections from Panama and Curacao, 19 crude extracts and 301 fractions were sent to PDSP for CNS screening. Due to sample size requirements not all fractions created are sent for screening, resulting in a lower number of total fractions screened compared to the theoretical amount of 370 fractions and crudes. In this screen, any fraction that displays greater than 50% inhibition of radioligand binding to a receptor is considered a “hit” at that receptor. Data analysis of results from these screens is presented herein.

### 2.2. Analysis of Crude Extracts

**Figure 2:**
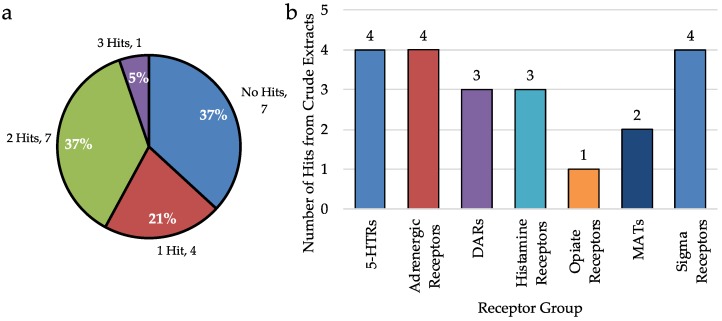
Analysis of biological data from *only* the crude extracts, before fractionation. (**a**) Analysis of the number of hits per crude extract. Few hits are detected when only the crude extracts are analyzed. (**b**) Number of hits detected from crude extracts at selected receptor groups.

First, to evaluate the effect of pre-fractionation on hit rate, the binding data of only the crude extracts was analyzed. When considering the 19 crude extracts only 2.9% of assays run result in a hit. Of the 19 extracts, seven had no hits, four had just one hit, seven had two hits, and only one extract had three hits (Figure 2a). Of these hits, only four were at 5-HTRs, three were at DARs, two were at MATs and there were four hits seen at σRs (Figure 2b). Although this data suggests that without pre-fractionation, 63% of extracts will result in a hit of some type, within the extract, there are few compounds in high enough concentration to be detected. Additionally, it is possible that any hit at from a crude is the result of multiple ligands which bind the receptor rather than one potent compound. 

### 2.3. Analysis of Hits from Individual Fractions

**Figure 3:**
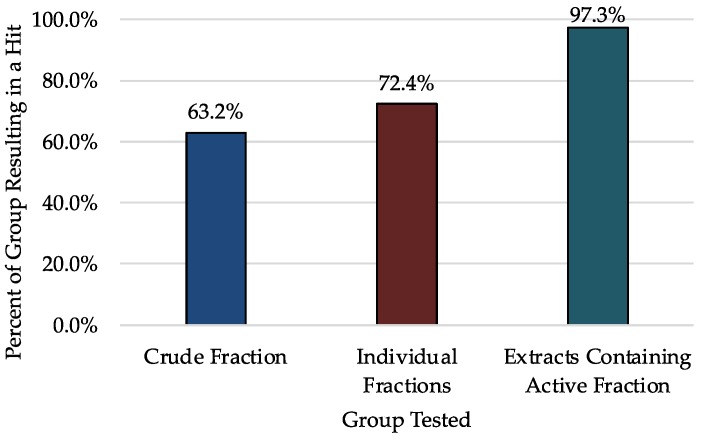
Comparison of hit rates for each extract with or without pre-fractionation. While 63.2% of crude extracts have at least 1 hit, 72.4% of fractions coming from these crude extracts result in a hit. Moreover, after-prefractionation, 97.3% of all extracts will produce a fraction with a hit.

Expanding the data analysis to include all 301 fractions tested, the screening effort resulted in 446 hits (3.8% overall hit rate) from 42 receptors. Of the 301 fractions, 215 were a hit at one or multiple receptors. While testing only the crude extracts would have resulted in 63% of cyanobacterial samples demonstrating some type of biological activity, when the extracts are pre-fractionated, the percent of active fractions increases to 72%. This is likely due to the elevated concentrations of individual compounds in each fraction compared to the pre-fractionated crude. To evaluate the impact of pre-fractionation, when individual fractions (A–I) are grouped together by parent extract, we see that 97% of cyanobacterial extracts will produce at least one fraction with a hit (Figure 3). Of the 37 cyanobacterial samples included in this study, only one sample contained no active fractions. Coincidentally, this extract had one of the lowest extract weights (60.7 mg) well below the average extract weight (2.5 g). Because concentrations of individual compounds are higher once fractionated, it is more likely that compounds present in lower concentrations in the crude will produce a hit at the fraction level rather than when the pre-fractionated extract is tested. These data confirm that the method of pre-fractionation before bioassay is important as it increases the percent of extracts demonstrating biological activity from roughly 2/3 to nearly 100%.

**Figure 4:**
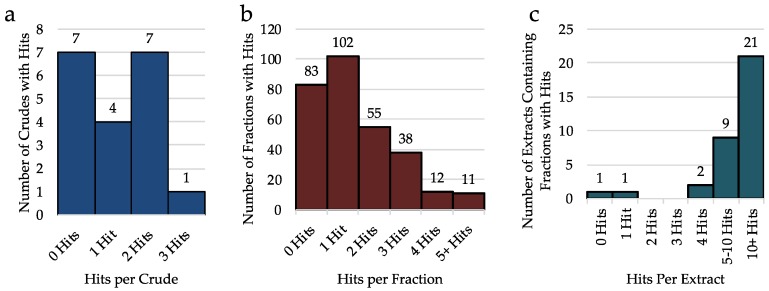
Number of hits per crude, fraction, or extracts once pre-fractionated. (**a**) Number of hits per crude extract (19 crude extracts total). (**b**) Number of hits per fraction (301 fractions total). (**c**) Number of hits per extract, once pre-fractionated (34 crude extracts total).

Moreover, while all crude extracts had 3 or fewer hits (Figure 4a), once fractionated, we see that the total number of hits from grouped fractions (A–I) from the same extract is almost always greater than 5 (Figure 4b), with most extracts having over 10 hits among their fractions (Figure 4c). This confirms that many of the biological activities were missed when only the crude was tested. Additionally, it is observed that most individual fractions produce 0, 1, or 2 hits, yet when grouping the nine fractions together, most extracts will have at least 10, this is because the extracts typically consist of fractions with 0 or 1 hits and then one or two fractions with a large number of hits (typically the G, H, or I fraction).

**Figure 5:**
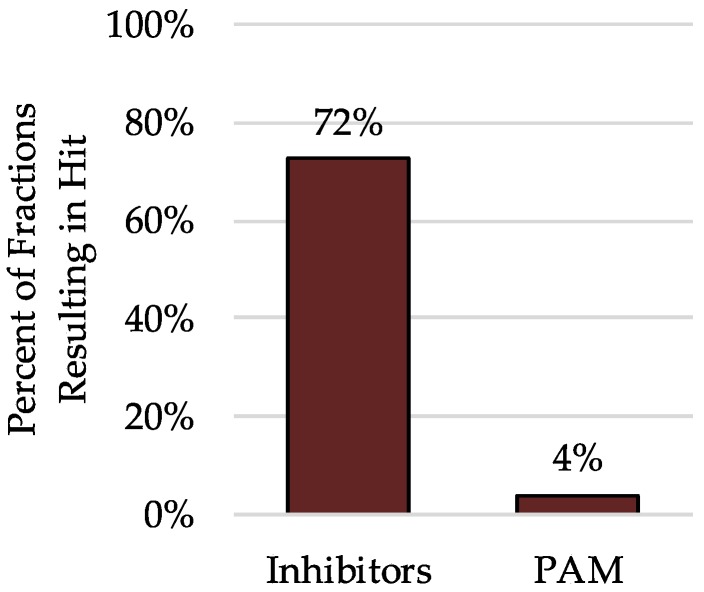
Percent of fractions resulting in a traditional hit (inhibits radioligand binding >50%), or a positive allosteric modulator (increases radioligand binding >50%).

In primary binding assays, some fractions showed an increase in radioligand binding rather than a decrease. Twelve of the 301 fractions increased radioligand binding by more than 50%, indicating the possible presence of a positive allosteric modulator (PAM) (Figure 5). While most fractions were a PAM for only one receptor, or a PAM for one receptor along with one or two inhibitor hits, one fraction increased radioligand binding by 50% at seven 5-HTRs, three adrenergic receptors, three DARs, one Histamine receptor and both the σ_1_R and σ_2_Rs. The increase in radioligand binding was as high as 731% in one case, suggesting the presence of a highly potent, non-selective PAM for GPCRs. Although these leads have not been further investigated, this data suggests that this primary screening process could be used to find selective, potent, positive allosteric modulators in addition to orthosteric agonists or antagonists.

### 2.4. Analysis of Hits by Receptor Group

**Figure 6:**
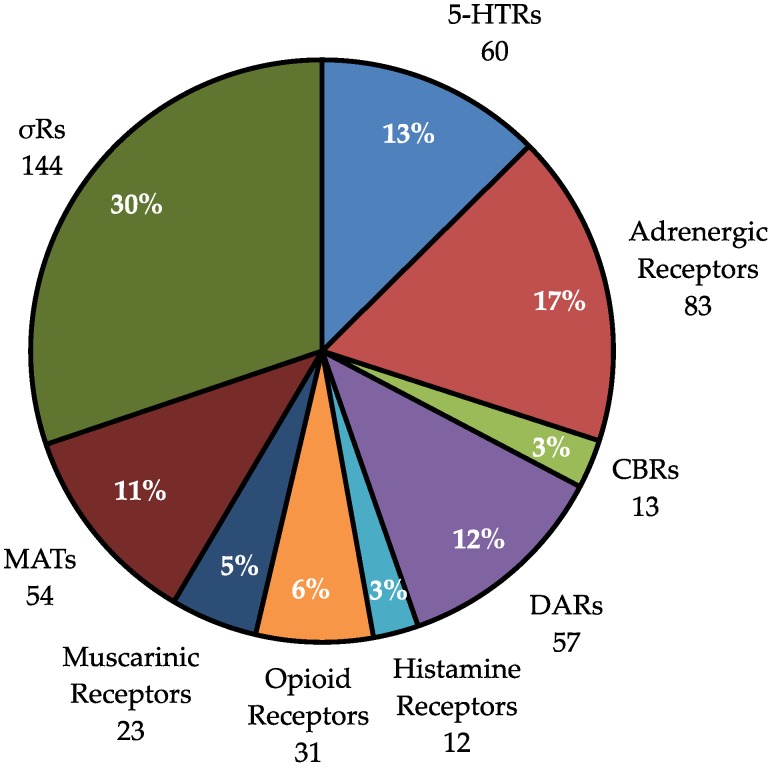
Percent of hits at each receptor group. (446 total hits, number of hits at each group is listed inside of graph).

Of the 446 hits produced by our 37 cyanobacterial samples, the most hits were produced at σRs (144 hits, 30%, Figure 6) followed by adrenergic receptors (83 hits, 17%), 5-HTRs (60 hits, 13%), DARs (57 hits, 12%), and MATs (54 hits, 11%). It is surprising that so many hits are seen at adrenergic receptors, as there are few published natural products that target these receptors; however, these data suggest that cyanobacteria may in fact be an underexplored source of adrenergic ligands. We see few hits at CBRs, histamine, AchRs and opiate receptors, these four groups combined comprise fewer than 20% of the hits.

#### 2.4.1. Analysis of Serotonin Receptor Hits

**Figure 7:**
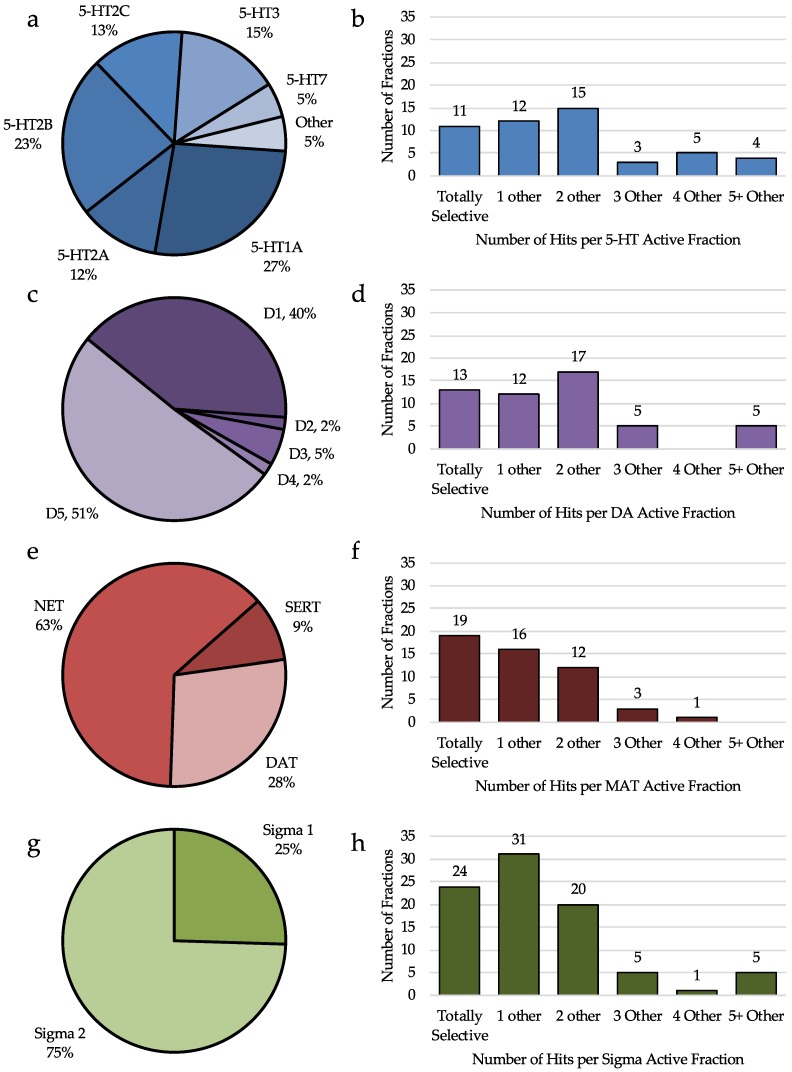
Analysis of hits by receptor subtype and selectivity of fractions by receptor group. (**a**) Percent of 5-HTR hits (60 hits total) at each receptor subtype. (**b**) Number of fractions with a 5-HT hit (50 total) having total selectivity (no other hits), one other hit, two other hits, etc. (**c**) Percent of DAR hits (57 hits total) at each receptor subtype. (**d**) Number of fractions with a DAR hit (52 total) having total selectivity (no other hits), one other hit, two other hits, etc. (**e**) Percent of MAT hits (54 hits total) at each receptor subtype. (**f**) Number of fractions with a MAT hit (51 total) having total selectivity (no other hits), one other hit, two other hits, etc. (**g**) Percent of σR hits (144 hits total) at each receptor subtype. (**h**) Number of fractions with a σR hit (86 total) having total selectivity (no other hits), one other hit, two other hits, etc.

Serotonin receptors have long been studied as a source of therapies for depression and anxiety, but more recently as therapies for pain. Analysis of our library revealed that 50 of the 301 fractions tested yielded at least one hit at a 5-HTR (Figure 7b). Of these fractions, 11 were totally selective for a single 5-HTR, showing no activity at other receptors. The majority of fractions with a hit at 5-HT, have two or fewer hits at other receptors, showing that this high throughput screen can provide leads for subtype selective ligands of 5-HTRs. Of 60 total hits at 5-HTRs, the most frequent hits were 5-HT_1A_, 5-HT_2B_, 5-HT_3_, and 5-HT_2C_ with 16, 14, nine, and eight hits, respectively (Figure 7b). The 5-HT_1A_ receptor specifically has been shown to be involved with anxiety, mood, and nociception, making it an attractive target for pain, depression, and anxiety. While there has been connections with 5-HT_2B_ and 5-HT_3_ and anxiety, neither is a target for pain. However the 5-HT_2C_ target has been connected to anxiety, depression and pain as well. Based on this analysis, at least 40% of 5-HT hits are at targets with mechanisms in pain, anxiety, and depression signaling.

#### 2.4.2. Analysis of Dopamine Receptor Hits

Dopamine, which is known to have roles in motor function and reward, among other roles, is now thought to be involved with anxiety, depression, and pain as well. Of the 57 DAR hits, produced by 52 different fractions, about 23% are totally selective for only one receptor, and another 21% have a hit at only one other receptor (Figure 7d). For the most part, our fractionation method has produced fractions with moderately selective activity at DARs. Interestingly 51% of the DAR hits bind to the D_5_ subtype and 40% bind the D_1_ subtype, which have both been linked to pain modulation. However, the D_2_R, D_3_R, and D_4_R, which have all been linked to anxiety and depression comprise less than 10% of the hits at DARs (Figure 7c). This suggests that the fractionation method used, is useful for finding leads for the treatment of pain through DARs, but not as useful for the treatment of anxiety or depression. This could be due to the nature of compounds produced by cyanobacteria, but it is possible that the fractionation method is not producing fractions with high enough concentrations of DA ligands to produce a hit at D_2_, D_3_, or D_4_ receptors.

#### 2.4.3. Analysis of Monoamine Transporter Hits

Serotonin and norepinerphrine transporters have been used to treat pain anxiety and depression in the form of SNRIs and SSRIs for years. There have been studies that also show the role of DATs in modulation of anxiety and depression, especially in Parkinson’s patients, however to date, there is not strong evidence to suggest any role of DATs in pain signaling. From the 446 hits detected from our fractions, 54 hits (11%) were at MATs (Figure 7f). Nineteen of the fractions with a MAT hit were totally selective over other receptors, and 16 were selective for only one other receptor, often in the corresponding neurotransmitter family. Of the 54 hits, only 9% were SERT hits. The majority of hits were NET hits (63%), and 28% of hits were DAT hits (Figure 7e). This suggest that of our MAT hits, 72% (SERT and NET) have the potential to affect pain, anxiety, and/or depression pathways, while the remaining 28% (DAT) could still affect anxiety and depression.

#### 2.4.4. Analysis of Sigma Receptor Hits

Both the σ_1_ and σ_2_ receptors have been shown to be involved in the neuromodulation of pain. While the σ_1_R has also been shown to be involved in depression and anxiety, this has not yet been demonstrated for the more recently discovered σ_2_R. Of the 446 hits detected in our screen, 144 of these hits are at σRs (Figure 7h). The overall hit rate for the σ_1_R is 9%, while the hit rate for σ_2_R is 26%. Suggesting that cyanobacteria may be a rich source of σR ligands. Of the fractions with a σR hit, 24 are totally selective for the σR, 31 have a hit at only one other fraction, and 20 have a hit at two other fractions. While 25% of the hits are at the σ_1_R, 75% bind to σ_2_R, and only four fractions have a hit at both σRs (Figure 7g). Because the role of σ_2_R in neuronal signaling is not as well understood, there is a need for ligands which are selective for σ_2_R over σ_1_R, and this analysis demonstrates that marine bacteria may be an abundant source for these compounds.

### 2.5. Analysis of Hits by Fraction

**Figure 8:**
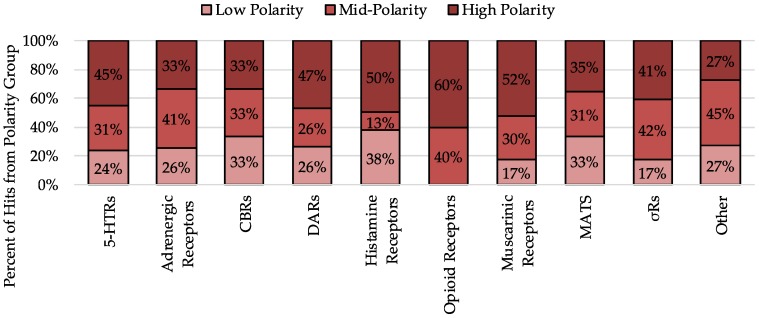
Percent of hits at each receptor group originating from low polarity (A, B, C), mid-polarity (D, E, F), and high polarity (G, H, I) fractions. Most hits come from high polarity fractions.

Next, the number of hits arising from low-polar (A, B, and C), mid-polar (D, E, and F), and highly-polar fractions (G, H, and I) was analyzed to understand the distribution of hits among the fractions we produce (Figure 8). For 5-HTRs and DARs, approximately 50% of hits come from the highly polar fractions (G, H, and I). Additionally, for MATs, the more of the hits come from highly polar compared to mid-polar or low-polar, however, it is a fairly even split. In general, the majority of any hits arise from highly polar fractions, and the fewest hits are seen with low-polar fractions. Interestingly hits at opiate receptors, which make up 6% of hits, only come from mid polar and highly polar fraction. Analyzing individual fractions within the polar groups revealed that while fractions G, H, and I all have over 50 hits arising from each fraction, fraction A had only 18 hits and B had 27 hits.

**Figure 9:**
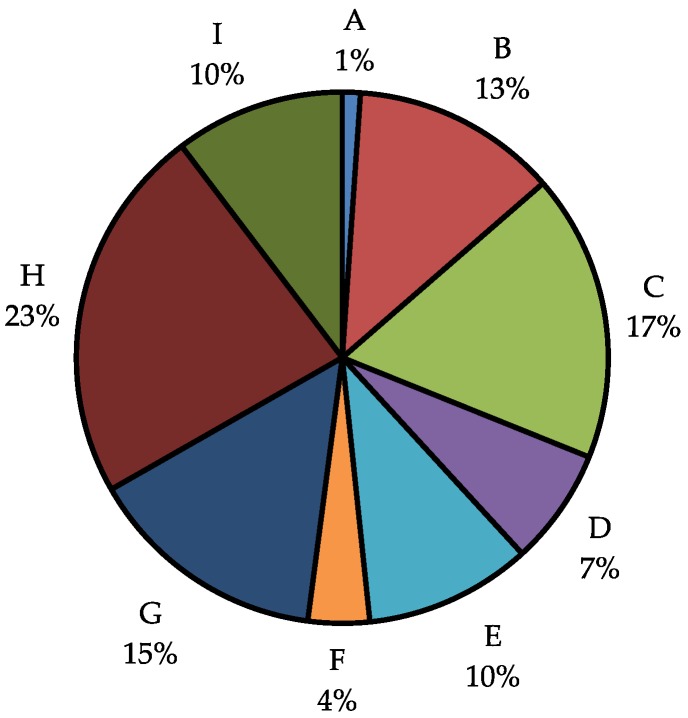
Average mass of each fraction. Fractions G, H, and I comprise almost 50% of the mass, while fractions A, D, and F comprise less than 15%.

This uneven distribution of hits among fractions prompted the analysis of the average mass of each fraction. It is common that the largest fraction from an extract is often G, H, or I. By adding the total mass of each fraction with other fractions of the same polarity, it was determined that the highly-polar fractions together (G, H and I) comprise almost 50% of the extract masses (Figure 9). Specifically, fraction H is on average 23% of extract mass. It is not surprising then, that the majority of our hits arise from these fractions, because they include most of the extract mass and likely most of the compounds.

**Figure 10:**
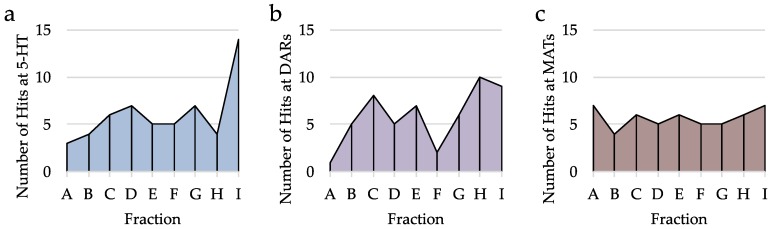
Analysis of number of hits by fraction. (**a**) Number of hits per fraction at 5-HTRs (60 hits total). (**b**) Number of hits per fraction at DARs (57 hits total). (**c**) Number of hits per fraction at MATs (54 hits total).

Next, the hit rate for each fraction among 5-HTRs, DARs, and MATs were analyzed more specifically. For 5-HTRs, it is seen that the most hits come from fraction I, followed by both G and H (Figure 10a). Interestingly, there is a dip in the number of hits at fraction H. Although there could be a true lack of 5-HT ligands at this polarity, it is more likely that the size of the H fraction is often too large to provide high enough concentrations of 5-HT ligands for the bioassay. With DAR hits, we see that both H and I have the greatest number of hits despite their large fraction mass (Figure 10b). On the contrary, MAT hits remain constant across all fractions ranging from four to five hits per fraction (Figure 10c). However, because of the uneven mass distribution, it’s possible that the highly polar fractions also have MAT ligands that go undetected due to low concentrations in the large fractions This data suggests that despite the large mass of the highly polar fractions, these still produce the most hits at the targeted receptors.

## 3. Case Studies from Panamanian Cyanobacterial Extract DUQ0008

Presented herein are two case studies from a single extract which demonstrate the process of pre-screening to select fractions with desired activity. In the first example, a fraction was selected with affinity for the sigma 2 receptor. After further fractionation it lead to the isolation of a pure compound that retained activity at the same receptor. In the second example, four compounds were isolated from a fraction with affinity for 5-HTRs, however, the pure compounds did not possess the same activity as the parent fraction. These case studies are used to demonstrate that although this method can work as intended, the major compounds present will not always be the active molecules. Additional screening for affinity, efficacy, and specificity is required of any “hits” before further development can be undertaken. Additionally, multiple compounds within the parent fraction could be acting in an additive, antagonistic, or synergistic way further complicating discovery efforts.

A cyanobacterial biomass collected at Isla Mina, Panama was extracted exhaustively with CH_2_Cl_2_-MeOH (2:1) to afford 3.3 g of crude extract given the identification code DUQ0008. This extract was fractionated to yield nine fractions (A–I). The crude extract, along with the nine fractions were submitted to PDSP for screening. Fractions that resulted in a hit were then subjected to secondary screening using radioligand displacement assays to determine the IC_50_ value (Table 1).

Similar to the aforementioned trends, the low-polar fractions had fewer hits than the highly polar fractions. The majority of hits arose from fractions G, H, and I. With the goal of isolating compounds with activity at 5-HTR, DAR, MATs or σ_2_R, fractions C and G were selected for further study. While fraction G lead to the isolation of known cyclic depsipeptide veraguamide C (**1**), which had moderate and selective affinity for the σ_2_R, the fatty acid compounds **2**–**4** isolated from fraction C displayed no activity at the 5-HT_2C_ or D_1_ receptor. Fraction I is currently being studied to identify bioactive constituents. These results highlight that although CNS active compounds are present in cyanobacterial extracts and can be isolated and identified, the method by which they are isolated could be improved to aide in isolation of active compounds. 

### 3.1. Isolation and Biological Testing of Veraguamide C

**Figure 11:**
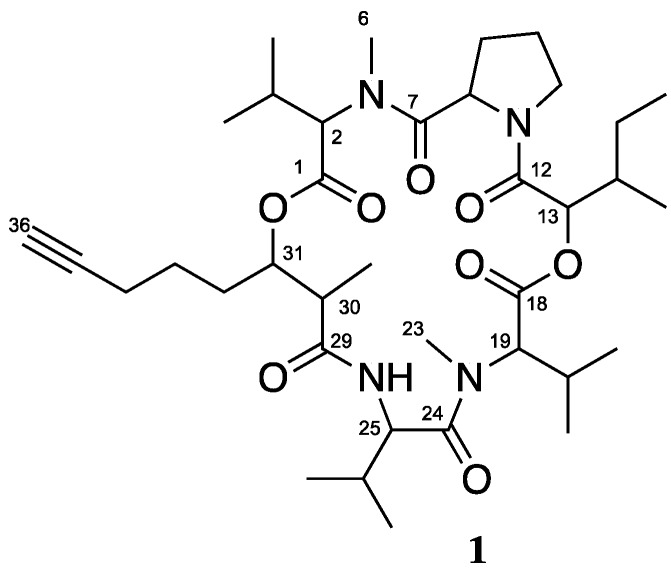
Structure of compound veraguamide C (**1**).

The fraction DUQ0008G (564.8 mg) demonstrated affinity to the 5-HT_2A_, 5-HT_2C_, Alpha_2B_, D_1_, D_5_, DOR and σ_2_R. The fraction was further purified using accelerated chromatographic isolation (ACI) and reversed-phase HPLC to yield 1.7 mg of known cyclic depsipeptide, veraguamide C (**1**, Figure 11). The structure was confirmed by comparison of experimental spectroscopic data (MS, ^1^H, ^13^C, COSY, HSQC, HMBC) with those in the literature [20]. Veraguamide C (**1**) was then resubmitted to PDSP for biological screening. While the parent fraction had IC_50_ = 1085.0 ng/mL +/−0.8 ng/mL at σ_2_R, compound **1** had K_i_ = 316.0 nM +/−0.8 nM at σ_2_R. Although veraguamide C showed greater than 50% inhibition of binding at 5-HT_2C_, the Ki was >10,000 nM reducing its likelihood as a true 5- HT_2C_ ligand. In this instance, lead identification through pre-fractionation and subsequent biological testing led to the isolation of a selective ligand for the σ_2_R. Further in vivo testing of this compound could be used to demonstrate its activity in pain models.

### 3.2. Isolation and Biological Testing of Fatty Acid Derivatives

**Figure 12:**
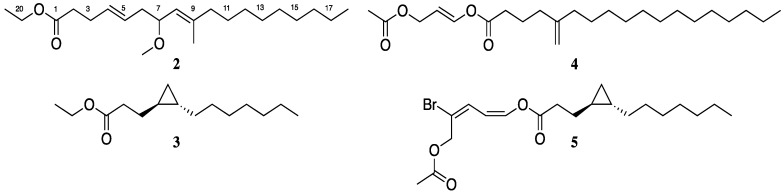
Structures of compounds **2**–**5** from 5-HT_2C_ active fraction.

The C fraction, which had activity at 5-HT_2C_ (191 ng/mL) and D_5_ (5083 ng/mL) was further purified using ACI and reversed-phase HPLC to yield 3.3 mg of Compound A (**2**, Figure 12). GCMS (Supplemental Figure S5) together with NMR data indicated the molecular formula C_22_H_40_O_3_ corresponding to three degrees of unsaturation. The ^1^H-NMR spectrum of **2** (Supplemental Figure S1) showed resonances assignable to four methyl groups, one of which (δ 1.26, H-22) through COSY (Supplemental Figure S2) correlation to the methylene at 4.13 (OCH_2_, q, *J* = 8.0, 7.9, H-21) was assigned to the ethyl group (substructure I) of the ester-linked diunsaturated fatty acid. The remaining methyl groups [δ 3.23 (OMe, s, H-20), 1.64 (s, H-19) and 0.88 (t, J = 7.1, H-18)], 2.34 (m, H-2), 2.32 (m, H-3), 2.28, 2.13 (2H, m, H-6), 2.01 (m, H-10), 1.41 (m, H-11) together with a 12H signal at δ 1.27 for 6 defined the substituted diunsaturated alkyl chain.

**Figure 13:**
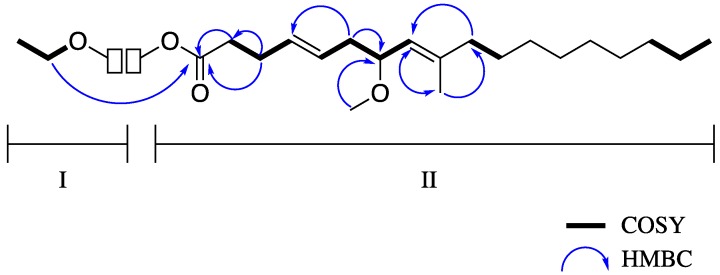
Selected COSY and HMBC correlations for compound **2** substructures I and II.

The ^13^C-NMR data derived from HSQC and HMBC experiments (Table 2 and Supplemental Figures S3 and S4), indicated the presence of four sp^2^ hybridized, and a carbonyl at δ 173.6 in addition to fifteen sp^3^ hybridized two of which were oxygen-bearing (δ 60.2 and 55.5). The complete assignment of all protons and carbons of compound **2** was achieved through the construction of the substructures (Figure 13) from 2D NMR data. In substructure II, the sequences of COSY correlations H-18–H-19–H-20 and H-10–H-11–H-12 linked the high intensity signal at δ 1.27 ppm (12 H) to the triplet at δ 0.88 ppm (H_3_-18) and the allylic methylene at δ 2.01 (t, *J* = 7.0, H-10) ppm, respectively. This suggested the presence of a saturated hydrocarbon moiety, typical of fatty acid derivatives.

Long range HMBC connectivities from the methylene hydrogens at H-10 and the methyl hydrogens at H-19 (δ 1.64 s) to the sp^2^ hybridized carbon at C-8 (δ 125.2), together with correlations of H-19 to C-10 (δ 27.8) linked the saturated hydrocarbon moiety, comprised of C-10–C-18, to the trisubstituted alkene of the fatty acid. The spin system formed by H-2, H-3, H-4, H-5, H-6, H-7, H-8 was assigned by COSY and ^2,3^*J* HMBC correlations. The chemical shifts for carbon at C-3 and C-4 were consistent with resonances for alkenes, while the chemical shift of the carbon at C-7 indicated that it is attached to oxygen. The presence of the singlet signal at δ 3.23 corresponding to the hydrogens of the methyl group at C-19 (δ 55.5), and HMBC correlations from these hydrogens to the oxygen-bearing sp^3^ carbon at C-7, confirmed placement of the methoxy group at position 7. HMBC correlations from the *α* and *β*-methylene hydrogens (H-2 and H-3 respectively) to the carbonyl at δ173.6 (C-1) completed the assignment of substructure II. Long range C–H correlations between methylene hydrogens and the methine resonances at δ 5.45 (2H, m, H-4, H-5), 4.99 (1H, d, *J* = 8.9, H-8), the hydrogens (δ 4.13, q, *J* = 8.0, 7.9) of the methylene group at C-18 to C-1 allowed for the combination of substructures I and II and the characterization of compound **2**.

Compound **3** was isolated as a colourless paste after purification by HPLC and was shown by ^1^H- and ^13^C-NMR data to be related to the C_13_ cyclopropyl fatty acid derivatives [18,21] isolated from marine cyanobacteria *Lyngbya majuscula* and includes grenadadiene (**5**), also isolated in this study. The structures of compounds **3**, **4** and **5** were confirmed by comparison of their spectroscopic data with those in the literature. To our knowledge this is the first report of the isolation of the putative precursor to the C_13_ cyclopropyl fatty acid derivatives isolated from marine cyanobacteria, from a natural source. Compound **3** was previously reported as an intermediate in the total synthesis, and determination of the absolute configuration of grenadamide [17]. It is difficult to ascertain whether compound **3** occurs naturally as the ethyl ester or the acid, as the original cyanobacterial biomass was preserved in ethanol prior to extraction. Fatty acids **2** and **3** along with the propenediester **4** were obtained in good yields for re-assay, but showed no significant binding affinity for 5-HTRs or any other receptor (inhibition at 10 mg/mL <50%, data not shown). As a result of the small quantities obtained, to probe the affinity of compound **5** for 5-HTRs re-isolation or small scale synthesis of this compound would have to be undertaken.

## 4. Conclusions

The data obtained from our screens summarized here indicate that compounds produced by marine cyanobacteria have the potential to modulate CNS signaling by targeting GPCRs and other receptors. Based on our library analysis, cyanobacteria are a rich source of compounds to modulate 5-HTRs, DARs, MATs, σRs, and adrenergic receptors. Additionally, many of the fractions generated in our lab from these cyanobacterial samples result in a hit at only 1 or two receptors, therefore, cyanobacteria are a potential source of *selective* ligands for these CNS receptors.

We have demonstrated that although CNS screening of crude extracts will still result in a hit for many samples, pre-fractionation before screening reveals new activities that are not observed when tested before fractionation. Additionally, we have analyzed the number of hits and mass arising from each fraction. This analysis revealed that the majority of our hits and mass are from the most polar G, H, and I fractions. Further analysis is underway to develop a new method for fractionation that would decrease the percent mass collected in G, H, and I, and enable the detection of lower concentration compounds present in these fractions.

We have demonstrated that the extraction and screening of fractions by PDSP can be an effective way to find lead fractions and isolate compounds with desired activity. So far our initial assessment of an extract with diverse activities among its fractions has yielded a σ_2_R active depsipeptide **1** and four fatty acid natural products **2**–**5**. This bioassay-guided study remains a useful tool to track bioactive metabolites from cyanobacterial fractions. Moreover, modifications to our fractionation method could be useful in increasing the number of hits detected in the highly polar range, and could decrease the number of steps required to isolate pure, bioactive compounds. Work is underway to develop an optimal method for pre-fractionation of cyanobacterial extracts for implementation with future cyanobacterial collections.

## 5. Materials and Methods

### 5.1. General Experimental Procedures

NMR spectra were recorded with chloroform as internal standard (δ_C_ 77.2, δ_H_ 7.26), on a Bruker 500 MHz spectrometer (Bruker Daltonics Inc., Billerica, MA, USA) operating at 499.7 MHz for ^1^H and 125.7 MHz for ^13^C equipped with a 5 mm PATXI ^1^HD/D-^13^C/^15^N Z-GRD Probe. GCMS was performed on Agilent Technologies 6890N GC system equipped with a 5973N mass selective detector (Agilent Technologies, Santa Clara, CA, USA). HPLC separation was carried out using a Dionex Ultimate 3000 pump system (Thermo Scientific, Waltham, MA, USA) with UV detection using HPLC grade solvents. Column chromatography was performed using Sorbent Technologies (Norcross, GA, USA) silica gel (230–400 mesh). Solvents were evaporated on a Heidolph (Schwabach, Germany) rotary evaporator.

### 5.2. Sample Material

Green/grey filamentous cyanobacteria were collected by hand on 29 January 2013 while snorkeling at a depth of 1 m off the coast of Isla Mina (GPS coordinates: N 8 29.717 W 78 59.947) in the Las Perlas Archipelago, Panama. The sample was fully suspended in ethanol:seawater (50:50) mixture to preserve the bacteria until it could be stored in a light-tight lab at −20 °C prior to extraction. A voucher specimen (PLP-29Jan13-3) is deposited in the Department of Medicinal Chemistry, Graduate School of Pharmacy, Duquesne University.

### 5.3. Extraction and Isolation

The cyanobacterial biomass (75 g, dry wt) was extracted exhaustively with 2:1 CH_2_Cl_2_-MeOH to afford 3.3 g of crude extract. This crude extract was fractionated over normal phase silica gel with a stepwise gradient solvent system of increasing polarity starting from 100% hexanes to 100% MeOH, to yield nine fractions (A–I).

The fraction eluting with 75% MeOH in EtOAc (fraction G, 564.8 mg) was subjected to fractionation on silica gel to give 7 fractions. The least polar fraction DUQ0008GF1 (52.3 mg) was subjected to semi-preparatory HPLC (Synergi-fusion 4μ, 150 × 10 mm column, Phenomenex, Torrance, CA, USA; 83% MeOH:H_2_O for 20 min, followed by 100% MeOH for 10 min; 1.5 mL/min). The peaks eluting between 25.0 and 30.0 min were combined (12.0 mg) then subjected to accelerated reverse phase chromatography (Snap KP-C18-HS, 12 g cartridge, Biotage, Uppsala, Sweden, 50% MeOH in 2.5 CV then 50–100% in 16 CV; 30 mL/min). The fraction collected at from 5.00 min to 5.75 min was concentrated *in vacuo* and determined to be known cyclic depsipeptide veraguamide C (**1**) (1.7 mg).

The fraction eluting with 40% EtOAc in hexanes (fraction C, 700 mg) was subjected to reversed-phase HPLC (Synergi-fusion 4μ, 150 × 10 mm) using a linear gradient of acetonitrile-H_2_O (40–100% acetonitrile in 30 min and then 100% acetonitrile for 10 min) to yield sub-fractions C2–4 corresponding to 101, 73.1 and 19.3 mg respectively. Sub-fraction C4 (19.3 mg) was further chromatographed on a Sep-Pak (Strata-X 33 μm polymeric reversed phase (10 mg/mL) tube), Phenomenex, Torrance, CA, USA, to yield compound **4**, 8 mg. Sub-fractions C2 and C3 were predominantly comprised of a white solid, which was not characterized. The interesting peaks observed in their ^1^H-NMR spectrum were compared to those of an earlier fraction, fraction B (eluted with 20% EtOAc in hexanes). A portion of the fraction B (147.7 mg) was chromatographed over silica gel (7.9 g) using EtOAc:hexane, gradient 2–5%. Combinations of 45 fractions, A–C, were obtained based upon TLC results (10% EtOAc:hexane). Subfraction A (79.7 mg) was subjected to reversed-phase HPLC (Synergi-fusion 4μ, 150 × 10 mm) using a linear gradient of acetonitrile-H_2_O (40–100% acetonitrile in 30 min and then 100% acetonitrile for 10 min) to yield compound **3** (1 mg, 15.47 mins). Subfraction B (12.7 mg) was subjected to reversed-phase HPLC (Synergi-fusion 4μ, 150 × 10 mm) using isocratic elution with 100% acetonitrile (30 mins) to yield compound **2** (1.3 mg) and compound **4** (5.2 mg). A second portion of fraction B (45.7 mg, eluted with 20% EtOAc in hexanes) was chromatographed using accelerated chromatographic isolation over snap KP sil (10 g) using a 7–100% EtOAc:hexane gradient to yield three fractions, A–C. Fraction A consisted of a mixture of compounds **4** and **5** (6 mg), as confirmed by ^1^H NMR. The mixture was further chromatographed using reversed-phase HPLC (Synergi-fusion 4μ, 150 × 10 mm) using isocratic elution with 100% acetonitrile (in 30 min) to yield compound **5** (1 mg). Compound **5** decomposed upon drying using air, 0.6 mg of compound **5** was retrieved under similar chromatographic conditions from the injection wash of fraction B (2.1 mg).

*Veraguamide C* (**1**): colourless oil; ^1^H-NMR (CDCl_3_, 500 MHz) δ 6.22 (1H, d, NH), 4.96 (1H, dd, H-8), 4.93 (1H, d, H-13), 4.88 (1H, d, H-31), 4.76 (1H, t, H-25), 4.25 (1H, d, H-19), 4.05 (1H, d, H-2), 3.84 (2H, q, H-11b), 3.63 (2H, q, H-11a), 3.19 (1H, m, H-30), 3.03 (3H, s, H-6), 2.94 (3H, s, H-23), 2.31-2.20 (5H, m, H-20,H-9b, H-34), 2.1-1.9 (7H, m, H-32b, H-3, H-10, H-14, H-36), 1.80-1.79 (3H, m, H-9a, H-26), 1.65–1.51 (6H, m, H-33a, H-15), 1.48-1.26 (5H, m, H-33b, H-37), 1.13-1.0(9H, m, H-22, H-21, H-17), 0.99-0.91 (9H, m, H-5, H-3, H-27), 0.89-0.8 (6H, m, H-28, H-16); ^13^C-NMR (CDCl_3_, 125 MHz): δ 173.5 (C24), 172.4 (C7), 171.0 (C1), 170.6 (C29), 169.8 (C18), 76.7 (C31), 76 (C13), 68.9 (C17), 66 (C19), 64 (C2), 57.3 (C8), 52.2 (C25), 47.2 (C11), 42.4 (C30), 38.5 (C26), 35.4 (C14), 30.3 (C23), 29.4 (C8), 28.8 (C6), 28.6 (C20), 27.6 (C32), 27.6 (C3), 25.2 (C33), 25.2 (C10), 24.7 (C15), 20.3 (C22), 20.3 (C21), 18.1 (C34), 16.4 (C27), 15.9 (C5), 15.9 (C4), 14.5 (C37), 13.85 (C17), 11.41 (C28), 11.09 (C16); ESIMS *m/z* = 733.98 calcd. for C_37_H_95_N_4_O_9_Na_2_ [M + 2Na − H]

*Compound***2**: colourless oil; ^1^H-, ^13^C- and 2D-NMR see Table 1; GCMS: 16.5 min, *m/z* = 237, 194, 101, 55.

*Compound***3**: colourless oil; ^1^H-NMR (CDCl_3_, 500 MHz) δ 4.18 (2H, q, *J* = 7.9, 7.9, H-14), 2.39 (2H, t, *J* = 7.7, H-2), 1.56 (2H, m, H-3), 1.35 (2H, m, H-11), 1.31 (2H, m, H-12), 1.28 (6H, m, H-8–10), 1.23 (1H, m, H-7), 1.14 (1H, m, H-7), 0.90 (3H, t, *J* = 6.8, H-13), 0.46 (1H, m, H-4), 0.46 (1H, m, H-6) 0.22 (2H, m, H-5); ^13^C-NMR (CDCl_3_, 125 MHz): δ 173.9 (C1), 60.3 (C14), 34.6 (C2), 34.2 (C7), 32.0 (C11), 29.6 (C3), 29.6 (C8-10), 22.7 (C12), 18.2 (C6), 18.0 (C4), 14.2 (C13), 14.2 (C15), 11.8 (C5); GCMS:11.3 min, *m/z* = 240, 194, 152.

*Propenediester* (**4**): colourless oil; ^1^H-NMR (CDCl_3_, 500 MHz) δ 7.25 (1H, dt, *J* = 6.5, 4.4, H-20), 5.10 (1H, dt, *J* = 6.7, 6.4, H-21), 4.78 (1H, s, H-19), 4.74 (1H, s, H-19), 4.75 (1H, d, *J* = 6.5, H-22), 2.45 (2H, t, *J* = 7.5, H-2), 2.09 (2H, t, *J* = 2.8, H-4), 2.09 (3H, s, H-24), 2.01 (2H, t, *J* = 7.6, H-6), 1.83 (2H, m, H-3), 1.43 (2H, m, H-7), 1.28 (22H, m, H-7–17); ^13^C-NMR (CDCl_3_, 125 MHz): δ 170.8 (C23), 170.1 (C1), 148.6 (C5), 137.0 (C20), 109.8 (C19), 107.5 (C21), 57.9 (C22), 35.8 (C6), 35.2 (C4), 33.3 (C2), 31.9 (C16), 29.7 (C8), 29.7–29.3 (C9-15), 27.8 (C7), 22.7 (C3), 22.7 (C7), 20.9 (C24), 14.1 (C18); GCMS: 20.5 min, *m/z* = 279, 110, 97.

*Grenadadiene* (**5**): colourless oil; ^1^H-NMR (CDCl_3_, 500 MHz) δ 7.32 (1H, d, *J* = 6.5, H-14), 7.00 (1H, d, *J* = 10.5, H-16), 5.75 (1H, dd, *J* = 10.5, 6.8, H-15), 4.86 (2H, s, H-18), 2.55 (2H, t, *J* = 7.7, H-2), 2.16 (3H, 2, H-20), 1.61 (2H, m, H-3), 1.34–1.28 (6H, m, H-8–10), 1.28 (2H, m, H-12), 1.27 (2H, m, H-11), 1.24 (1H, m, H-7), 1.15 (1H, m, H-7), 0.90 (3H, t, *J* = 6.8, H-13), 2.55 (2H, t, *J* = 7.7, H-2), 0.48 (1H, m, H-4), 0.48 (1H, m, H-6) 0.25 (2H, *J* = 6.7, H-5); ^13^C-NMR (CDCl_3_, 125 MHz): δ 170.4 (C19), 169.9 (C1), 137.8 (C14), 124.5 (C16), 121.0 (C17), 109.0 (C15), 69.1 (C18), 34.3 (C2), 34.0 (C7), 31.8 (C11), 29.8 (C8–10), 29.2 (C3), 22.8 (C12), 20.9 (C20), 19.1 (C6), 18.2 (C4), 14.2 (C13), 11.8 (C5); GCMS:18.5 min, *m*/*z* = 414 (^79^Br), 416 (^81^Br), 293, 281, 220 (^79^Br), 222 (^81^Br).

### 5.4. Receptor Binding Profile

IC_50_ determinations/receptor binding profiles were generously provided by the National Institute of Mental Health’s Psychoactive Drug Screening Program, Contract # HHSN-271-2018-00023-C (NIMH PDSP). The NIMH PDSP is Directed by Bryan L. Roth MD, PhD at the University of North Carolina at Chapel Hill and Project Officer Jamie Driscoll at NIMH, Bethesda MD, USA. Detailed information regarding the assay protocol can be found at http://pdspdb.unc.edu/pdspWeb/.

## Figures and Tables

**Table 1:** IC_50_ values of DUQ0008 crude extract and fractions at active receptors.

	5-HT_1A_ (IC_50_)	5-HT_2A_ (IC_50_)	5-HT_2C_ (IC_50_)	5-HT_7_ (IC_50_)	Alpha_2B_ (IC_50_)	D_1_ (IC_50_)	D_5_ (IC_50_)	DOR (IC_50_)	σ_2_R (IC_50_)
Crude	-	-	-	-	-	-	-	-	722.0 ± 0.8
A	-	-	-	-	-	-	-	-	-
B	-	-	-	-	-	-	-	-	-
C	-	-	191.0 ± 0.8	-	-	5083.0 ± 0.9	-	-	-
D	-	>10,000	-	-	1999.7 ± 0.8	-	-	1776.0 ± 0.9	-
E	-	-	-	-	-	-	-	-	1502.0 ± 0.8
F	-	-	-	-	-	-	-	-	-
G	-	1854.0 ± 0.9	98.0 ± 0.8	-	2030.0 ± 0.8	1526.0 ± 0.9	1531.0 ± 0.9	1618.0 ± 0.9	1085.0 ± 0.8
H	-	-	-	-	-	-	-	-	1874.0 ± 0.8
I	2441.0 ± 0.8	-	-	1223.00 ± 0.8	-	-	-	-	424.0 ± 0.8

Open boxes with - indicate that fractions failed primary binding criterion of >50% inhibition at 10 mg/mL. N = 3 separate determinations for each value. All data presented as ng/mL.

**Table 2:** NMR Spectroscopic data for Compound **2** in CDCl_3_.

Pos.	δ_C_, Type	δ_H_ Multi. (*J* in Hz)	HMBC *^a^*
1	173.6, C		
2	34.4, CH_2_	2.34 m	1, 3
3	28.0, CH_2_	2.32 m	1
4	127.4, CH	5.47 m	6
5	130.4, CH	5.45 m	6
6	38.9, CH_2_	2.28 m, 2.13 m	4, 5, 7
7	77.2, CH	3.88 m	20
8	125.2, CH	4.99 d (8.9)	10, 19
9	140.2, C		
10	39.9, CH_2_	2.01 t (7.0)	8, 9, 11, 19
11	27.8, CH_2_	1.41 m	
12	29.4, CH_2_	1.27 m	
13	29.4, CH_2_	1.27 m	
14	29.4, CH_2_	1.27 m	
15	29.4, CH_2_	1.27 m	
16	31.9, CH_2_	1.27 m	
17	22.5, CH_2_	1.29 m *^b^*	
18	14.4, CH_3_	0.88 t (7.1)	16, 17
19	16.7, CH_3_	1.64 s	8, 9, 10
20	55.5, OCH_3_	3.23 s	7
21	60.2, OCH_2_	4.13 q (8.0, 7.9)	1, 22
22	14.5, CH_3_	1.26 t *^b^*	21

*^a^* Carbon correlating to proton shift, *^b^* Signal partially obscured.

## Supplementary Materials

The following are available online. Figure S1: 1H NMR spectrum of compound **2** in CDCl3 at 500 MHz, Figure S2: COSY spectrum of compound **2** in CDCl3 at 500 MHz, Figure S3: HSQC spectrum of compound **2** in CDCl3 at 500 MHz, Figure S4: HMBC spectrum of compound 2 in CDCl3 at 500 MHz, Figure S5: GCMS spectrum of **2**.
