# Supplementary material for: Evaluating Marine Cyanobacteria as a Source for CNS Receptor Ligands
Source: Molecules. 2018 Oct 17;23(10):2665. doi: 10.3390/molecules23102665 (PMC6222545; doi:10.3390/molecules23102665)
Supplement: Supplementary file 1 [file molecules-23-02665-s001.pdf]

Evaluating Marine Cyanobacteria as a Source for CNS Receptor Ligands

Andrea L. Rague, Stacy-Ann J. Parker, and Kevin J. Tidgewell

Department of Medicinal Chemistry, Mylan School of Pharmacy, Duquesne University, Pittsburgh, PA 15282, United States

Contents of Supporting Information

**Contents**	**page**
**Figure S1**. ^{1}H NMR spectrum of compound **2** in $CDCl_3$ at 500 MHz	**2**
**Figure S2**. COSY spectrum of compound **2** in $CDCl_3$ at 500 MHz	**3**
**Figure S3**. HSQC spectrum of compound **2** in $CDCl_3$ at 500 MHz	**4**
**Figure S4**. HMBC spectrum of compound **2** in $CDCl_3$ at 500 MHz	**5**
**Figure S5**. GCMS spectrum of **2**	**6**

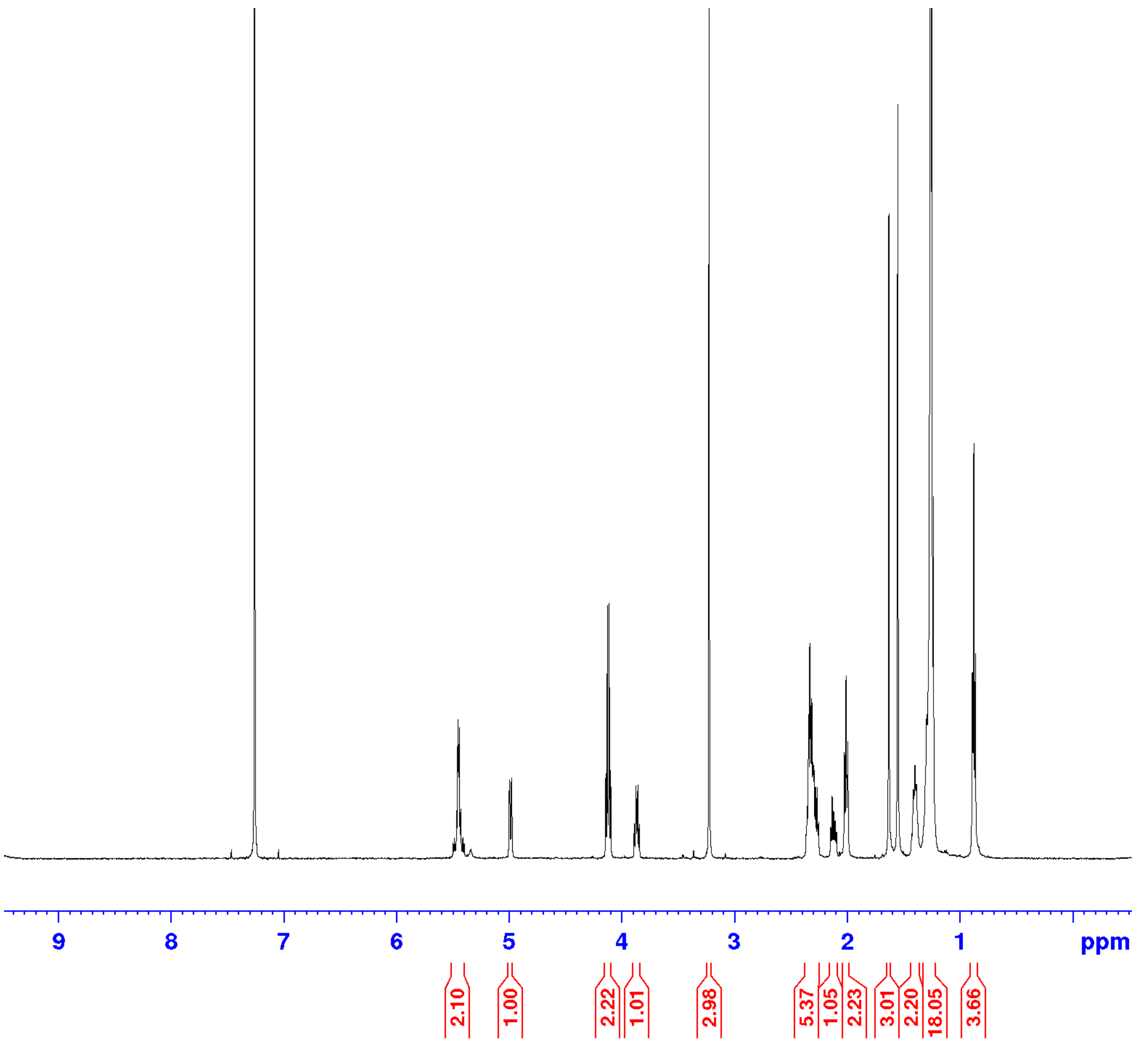

Figure S1. ^{1}H NMR spectrum of compound **2** in $CDCl_3$ at 500 MHz

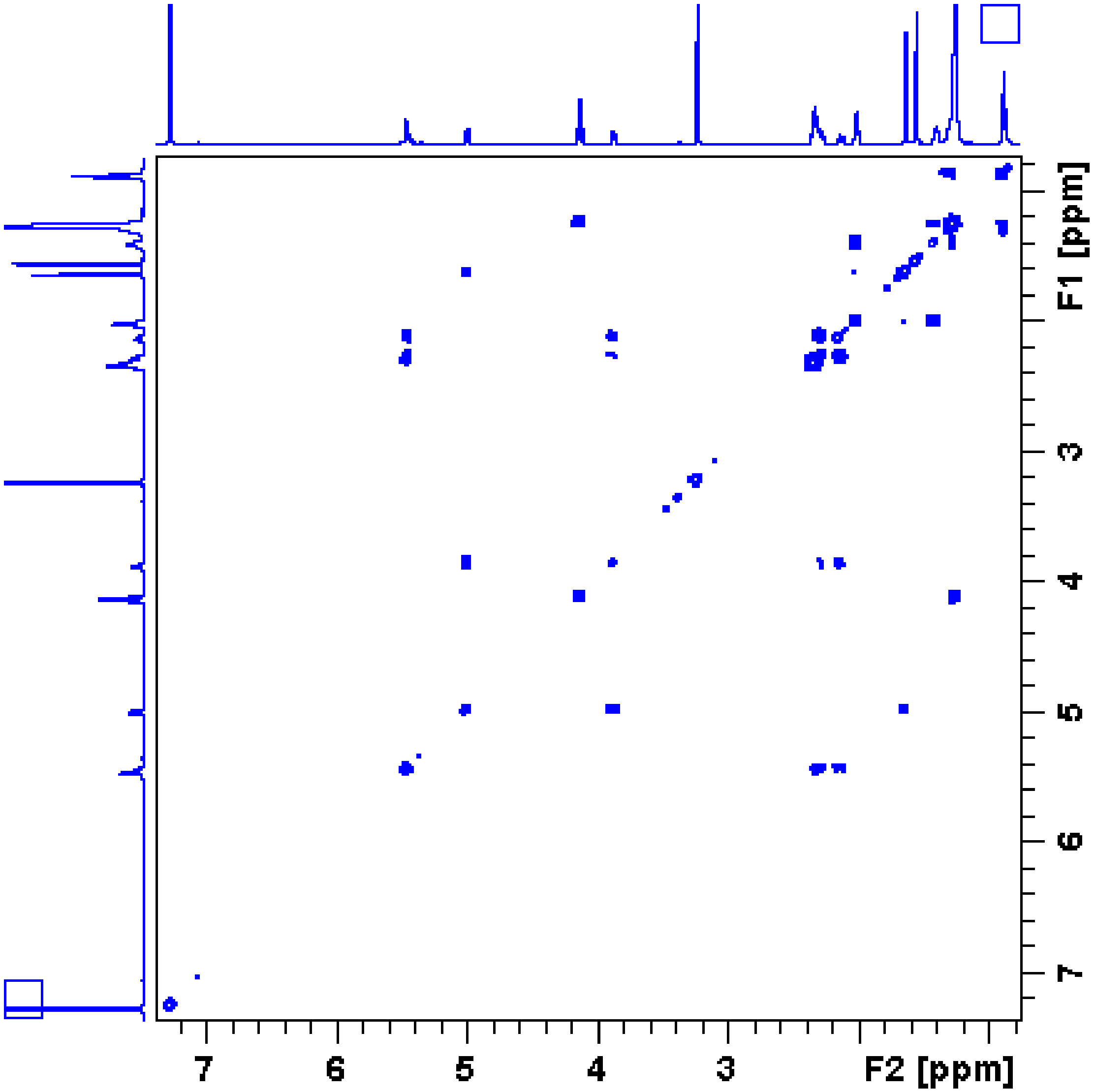

Figure S2. COSY spectrum of compound **2** in $CDCl_3$ at 500 MHz

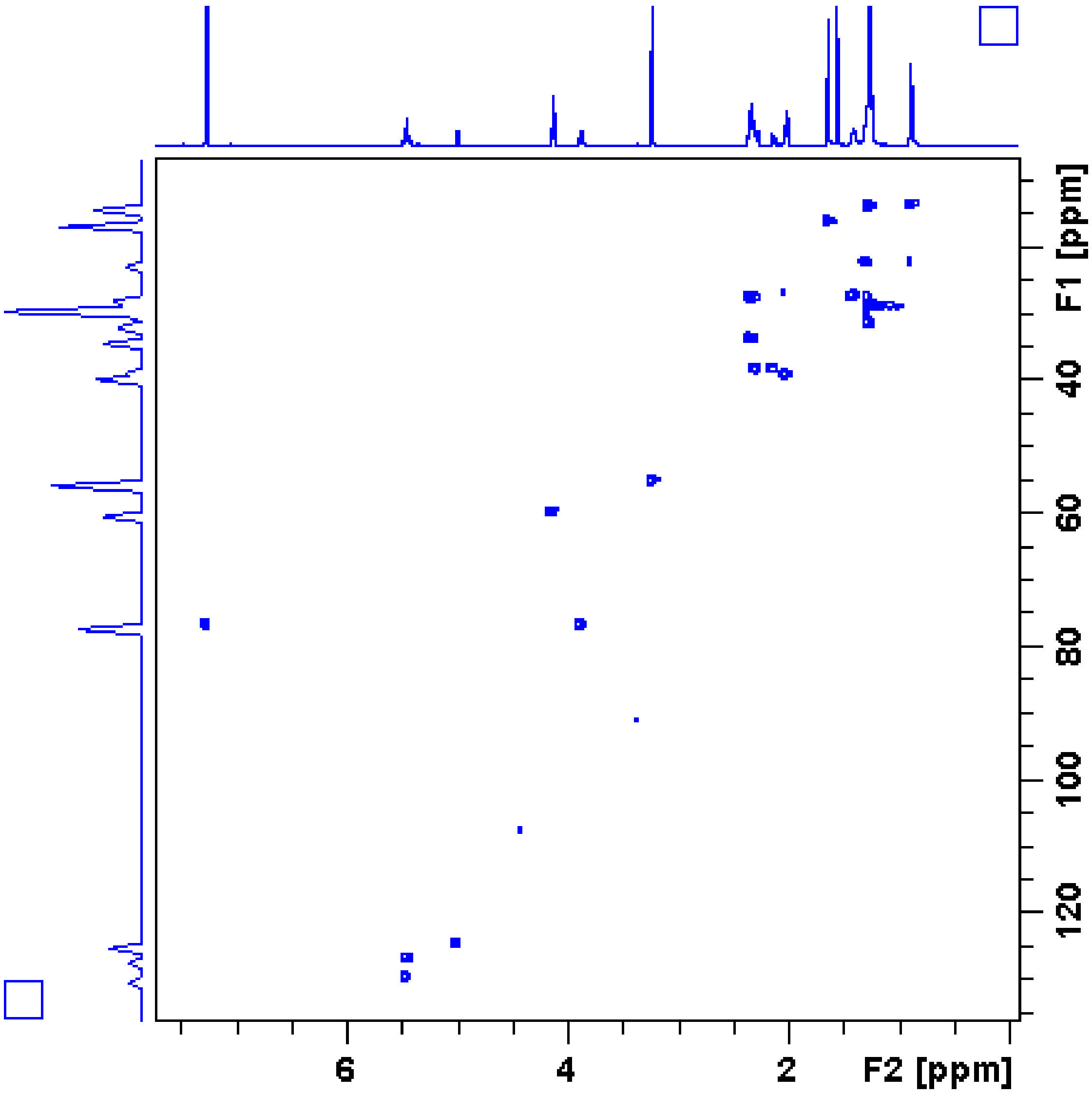

Figure S3. HSQC spectrum of compound **2** in $CDCl_3$ at 500 MHz

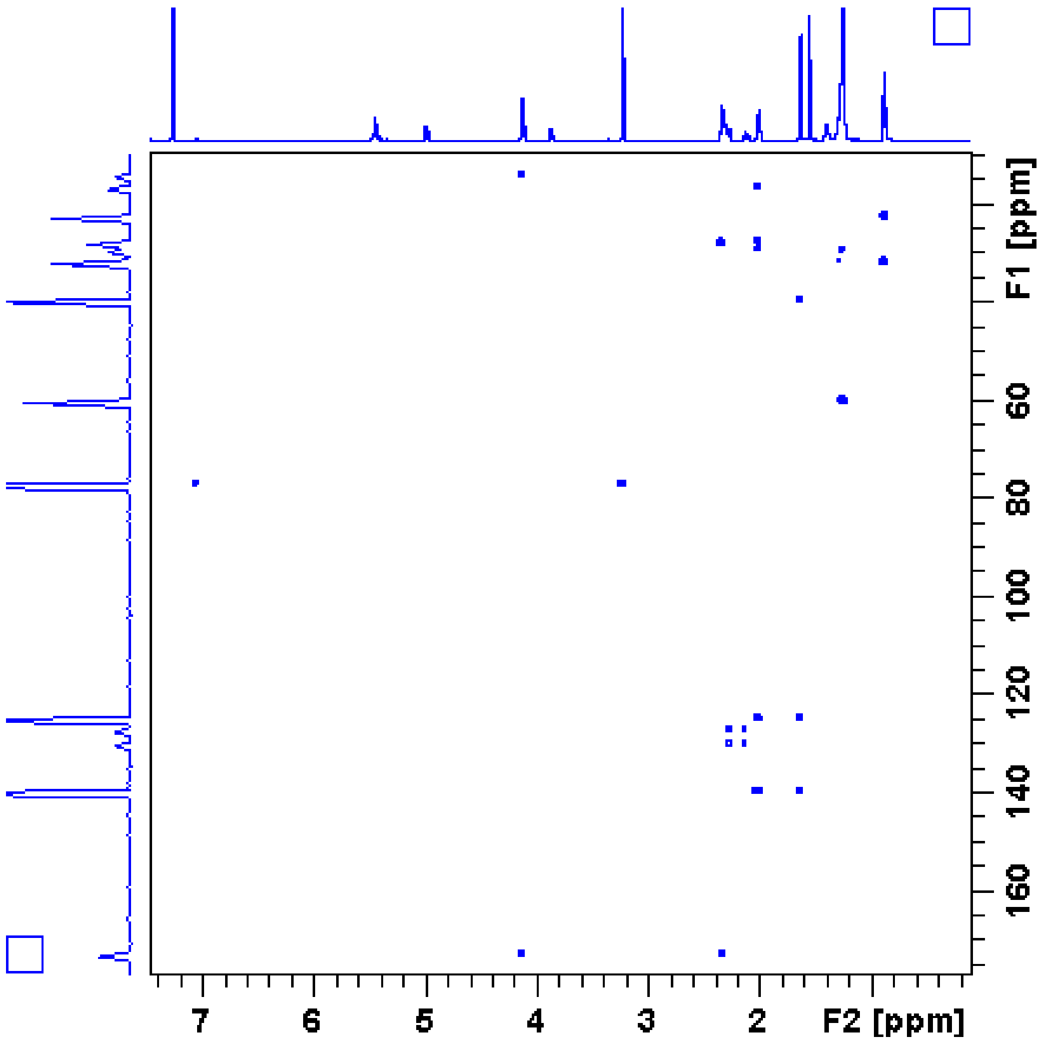

Figure S4. HMBC spectrum of compound **2** in $CDCl_3$ at 500 MHz

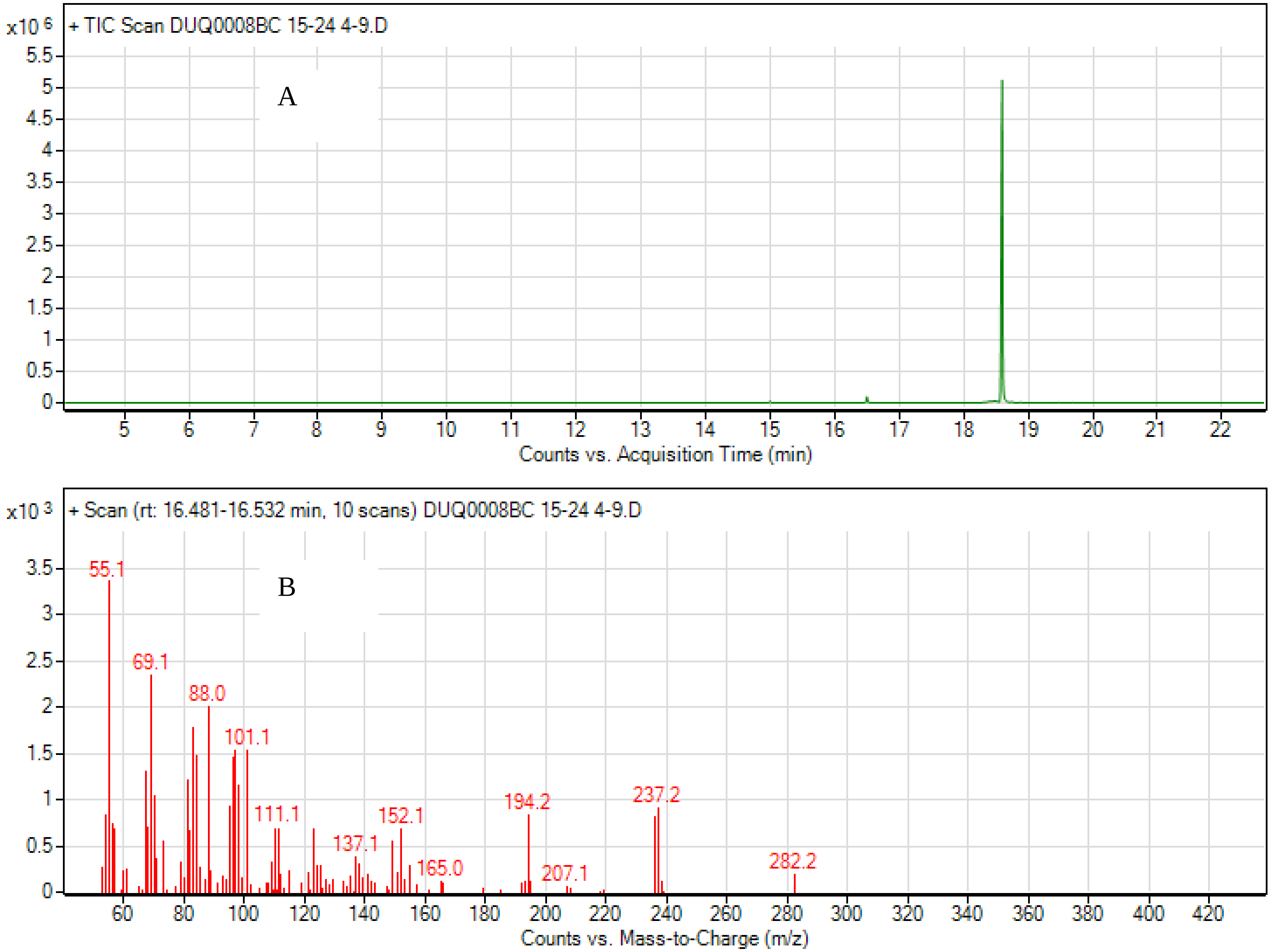

Figure S5. GC-MS chromatogram and mass spectrum of compound **2**. (A) TIC, total ion chromatogram, of **1** and (B) mass spectrum of compound **2**.
